# The mitochondria-targeted antioxidant MitoQ, attenuates exercise-induced mitochondrial DNA damage

**DOI:** 10.1016/j.redox.2020.101673

**Published:** 2020-08-06

**Authors:** Josh Williamson, Ciara M. Hughes, James N. Cobley, Gareth W. Davison

**Affiliations:** a Ulster University, Sport and Exercise Research Institute, Newtownabbey, Northern Ireland, UK; b Ulster University, Nursing and Health Research Institute, Newtownabbey, Northern Ireland, UK; c Free Radical Research Group, University of the Highlands and Islands, Centre for Health Sciences, Inverness, IV2 3JH, UK

**Keywords:** Comet assay, Oxidative stress, Mitochondria, Exercise, ROS, DNA

## Abstract

High-intensity exercise damages mitochondrial DNA (mtDNA) in skeletal muscle. Whether MitoQ - a redox active mitochondrial targeted quinone - can reduce exercise-induced mtDNA damage is unknown. In a double-blind, randomized, placebo-controlled design, twenty-four healthy male participants consisting of two groups (placebo; *n* = 12, MitoQ; *n* = 12) performed an exercise trial of 4 x 4-min bouts at 90–95% of heart rate max. Participants completed an acute (20 mg MitoQ or placebo 1-h pre-exercise) and chronic (21 days of supplementation) phase. Blood and skeletal muscle were sampled immediately pre- and post-exercise and analysed for nuclear and mtDNA damage, lipid hydroperoxides, lipid soluble antioxidants, and the ascorbyl free radical. Exercise significantly increased nuclear and mtDNA damage across lymphocytes and muscle (*P* < 0.05), which was accompanied with changes in lipid hydroperoxides, ascorbyl free radical, and α-tocopherol (*P* < 0.05). Acute MitoQ treatment failed to impact any biomarker likely due to insufficient initial bioavailability. However, chronic MitoQ treatment attenuated nuclear (*P* < 0.05) and mtDNA damage in lymphocytes and muscle tissue (*P* < 0.05). Our work is the first to show a protective effect of chronic MitoQ supplementation on the mitochondrial and nuclear genomes in lymphocytes and human muscle tissue following exercise, which is important for genome stability.

## Highlights

• Exercise damages mitochondrial DNA in lymphocytes and muscle tissue.
• Acute MitoQ ingestion has no impact on biomarkers of oxidative stress.
• Chronic MitoQ supplementation protects mitochondrial and nuclear DNA.

## Graphical abstract

**Figure:**
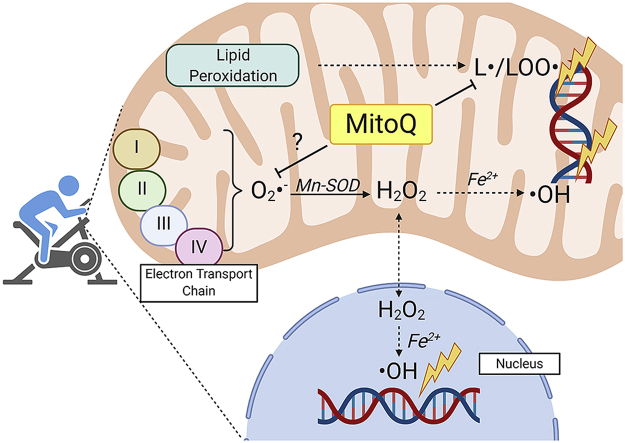

## 1 Introduction

Mitochondria have an established role in the life cycle of a cell, contributing to cellular networks aligned to metabolism, biosynthetic pathways, and apoptotic cell death [1]. Although the relationship between mitochondrial dysfunction and disease is complex, and the associated underlying mechanisms are still being investigated [2], a common denominator across a multitude of pathologies is an increased generation of reactive species, and subsequent mtDNA damage [3]. Mitochondrial DNA damage impairs bioenergetics, apoptosis, cell proliferation, and in turn, increases the likelihood of compromised organ function and pathology [4,5].

Exercise increases the univalent reduction of ground state molecular dioxygen (O_2_) to superoxide (O_2_·^-^) in skeletal muscle. NADPH oxidase enzymes are thought to dominate exercise-induced O_2_·- production, in part, because several factors (notably ATP demand) should decrease mitochondrial O_2_·^-^ production. Intriguingly, while net mitochondrial O_2_·^-^ production is decreased, the flavin mononucleotide of complex I continues to produce O_2_·^-^ in a metabolic milieu mimicking exercise in isolated mitochondria [6]. This observation combined with the potential for significant mitochondrial O_2_·- production in the minutes and hours after acute exercise, and their ability to act as a hydrogen peroxide (H_2_O_2_) sink, helps to reconcile our previous finding that exercise increases mtDNA damage (specifically 8-hydroxy-2-deoxyguanosine) [7]. Mechanistically, H_2_O_2_ potentiates proximal hydroxyl radical production by reacting with transition metals (e.g., Cu^+^) bound to DNA; thus, initiating the downstream generation of various oxidation products including, 8-hydroxy-2-deoxyguanosine. Presence of this commonly quantified base adduct following exercise is indicative of hydroxyl radical addition to guanine's eighth position, as guanine is the most readily oxidised base [8]. To date, the literature surrounding exercise-induced DNA damage has focused on nuclear DNA damage [9]; however, the lack of complex chromatin organisation and histone proteins [10,11], the accumulation of vicinal transition metal ions (e.g., ferrous iron), and the potential activation of secondary DNA- and lipid-oxidation products collectively make mtDNA more susceptible to oxidative attack compared to nuclear DNA [12,13]. Understanding and manipulating exercise-induced mtDNA damage is important for avoiding mtDNA heteroplasmy and maintaining genome integrity [14]. Interest in curtailing mtDNA damage has led to the development of several mitochondria targeted antioxidants including: MitoE [15], tiron [[16], [17], [18]], and MitoC [19].

One such therapeutic compound is the orally-available mitochondrial-targeted coenzyme Q10, termed Mitoquinone (MitoQ) [20]. Accumulation of coenzyme Q10 within mitochondria is limited by high lipophilicity, large molecular weight, and poor aqueous solubility, and as a result clinical trials often administer high doses [21]. Conversely, a triphenylphosphonium cation enables MitoQ to accumulate in mitochondria at approximately 100–1000 times greater than non-targeted derivatives [22]. Lipophilic cations increase their accumulation 10-fold for every 61.5 mV of membrane potential in accordance with the Nernst equation; this effective uptake is also supported by the plasma membrane potential [23]. Once absorbed, MitoQ is primarily reduced by complex II (but also glyceraldehyde 3-dehydrogenase in some tissues) to ubiquinol [24,25], which can act as a chain breaking antioxidant, and regenerate the alpha-tocopherol radical (ROO· + α-TOH → ROOH + α-TOH· [*k* = 10^5^ - 10^6^ M^−1^s^−1^]; α-TOH· + RH (PUFA) → α-TOH + R· (Alkyl Radical) [*k* = 1 × 10^−1^ M^−1^s^−1^]). Accordingly, MitoQ attenuates lipid peroxidation in isolated mitochondria [24,26] and peroxynitrite mediated oxidative damage [25]. Other protective effects of MitoQ have been observed in clinical applications including cardiac ischemia/reperfusion injury [27], chronic nitroglycerin exposure [28,29], sepsis [30], and liver damage [31], solidifying MitoQ as an attractive compound with potential therapeutic treatment for certain human diseases.

The majority (if not all) of exercise studies have used pleiotropic, non-selective antioxidants with unknown tissue distribution and quantification in an attempt to infer mechanistic conclusions relating to redox signalling from oxidative stress biomarkers [32]. Exercise-based research is clearly warranted to unambiguously clarify a regulatory mechanism between redox molecular modifications and a physiological outcome after administration of a targeted antioxidant [33]. Recently, Pham and colleagues (2020) demonstrated the ability of chronic MitoQ supplementation (20 mg/day) to suppress mitochondrial H_2_O_2_ release *ex vivo* and increase the expression of several enzymatic antioxidants. While their work adds to current understanding, whether MitoQ can impact functionally and translationally important sentinels of mtDNA damage is unknown. Accordingly, we aimed to determine whether: (1) a bout of high-intensity intermittent exercise (HIIE) damaged mtDNA; and (2) MitoQ could prevent mtDNA damage.

## 2 Materials and methods

### 2.1 Participants

Twenty-four (*n* = 24) apparently healthy, recreationally active males (age 25 ± 4 years, stature 181 ± 4 cm, mass 87 ± 11 kg) volunteered and subsequently provided their medical history prior to written informed consent. All participants were non-smokers and free from any form of medication or antioxidant supplementation for 4-weeks prior to, and throughout the study. The study was conducted in accordance with the Declaration of Helsinki and approved by a local University Ethics Committee.

### 2.2 Acute and chronic supplemention

**Fig. 1:**
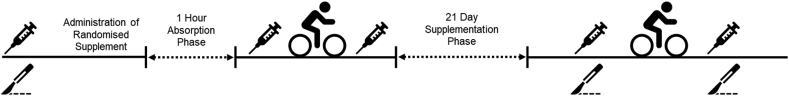
Schematic overview of experimental design. Participants remained in their assigned groups for the acute phase, and for 21 supplemental days of the chronic phase.

In a double-blind, randomized, placebo-controlled design, participants were allocated to two groups: MitoQ (*n* = 12) and placebo (*n* = 12), and subsequently took part in a two-phased supplementation trial. For the acute phase, either 20 mg MitoQ (Antipodean Pharmaceuticals; CA, USA), or placebo (Antipodean Pharmaceuticals [microcrystalline cellulose, tapioca, silicon dioxide]) excluding the active ingredient was consumed 1-h pre-exercise. MitoQ dosing was informed by previous human research [35,36]. Following HIIE, participants continued to supplement in their respective groups for 21-days (chronic phase). A schematic overview of the experimental trial is depicted in Fig. 1. Participants were instructed to consume MitoQ in a fasted state to maximise absorption based on known pharmacokinetic data (personal communication Prof. Michael Murphy, University of Cambridge).

### 2.3 High-intensity intermittent exercise

For all experimental testing, participants were required to complete a standardised 12-h overnight fast, and to refrain from exercise and alcohol for 48-h prior to testing. Following the familiarisation phase, participants completed an incremental test to exhaustion to determine maximum heart rate (HR_max_). Participants cycled at a cadence of 70–90 revolutions per minute on a friction-braked cycle ergometer (Monark, Sweden) to produce a power output equivalent to their bodyweight (1 W/kg). Workload was increased by 0.5 W/kg of body weight every 2 min until the participant could no longer maintain the required work rate [37].

Following the incremental exercise test to exhaustion, participants completed HIIE consisting of 4 x 4-min bouts. Each 4-min work interval corresponded to 90–95% of HR_max_ with a 3-min active recovery at 70% of HR_max_ [38]. Continuous heart rate (HR) monitoring was achieved via a portable short-angle telemetry device (Polar, Finland). Participants could drink water *ad libitum.*

### 2.4 Haematology and muscle tissue sampling

Blood was sampled from a prominent antecubital forearm vein pre-supplementation, post-acute supplementation (pre-exercise) and immediately post-exercise for both the acute and chronic phases. Peripheral blood mononuclear cells (PBMCs) were isolated by layering 3 ml of whole blood onto 3 ml of Histopaque-1077 (Sigma-Aldrich, St. Louis, MO), and centrifuged at 3500 rpm for 30 min at 4 °C. All blood was extracted using the vacutainer method, and subsequently centrifuged, aliquoted, and stored at −80 °C prior to biochemical analysis. An exercise-induced haemoconcentration was determined using the equations of Dill and Costill [39], incorporating haemoglobin and haematocrit indices. Packed cell volume (%) was measured using the microcapillary reader technique and corrected by 1.5% for plasma trapped within erythrocytes [40].

A randomized subsample of participants provided skeletal muscle tissue (MitoQ, *n* = 5; Placebo, *n* = 5), extracted at baseline, and pre- and post-exercise time points in chronic phase. Briefly, following local anesthetic (2% lidocaine), a small incision was made using a single-use sterile scalpel (Swan-Morton, Sheffield, England). The biopsy device (Acecut biopsy needle, TSK laboratories, Soja, Japan) was subsequently inserted at a 90-degree angle to the skin edge, and triggered to capture the muscle sample. The same investigator extracted all muscle tissue samples. Once collected, samples were immediately flash frozen in liquid nitrogen and stored at −80 °C until subsequent analysis.

### 2.5 Global mitochondrial DNA damage

A Long Amplicon-Quantitative Polymerase Chain Reaction (LA-qPCR) assay was utilised to determine total mtDNA damage. Total DNA was extracted from lymphocytes, human muscle tissue, and mouse C2C12 myoblasts using a Qiagen Genomic-Tip kit as previously outlined by Hunter et al. [41] and Furda et al. [42]. DNA quality and purity were quantified using a Nanodrop 2000 (Thermo Scientific, USA) spectrometry method (A_260_/A_280_ ≥ 1.85).

DNA was quantified using PicoGreen as per manufacturers instructions, and flouresence was measured with a 485 nm excitation filter and a 530 nm emission filter. Lambda DNA/HindIII was used to construct a standard curve to determine the concentration of unknown samples. All DNA samples were stored in TE buffer (10 mM Tris/1 mM EDTA) at 4 °C.

50 μl reactions were prepared by combining the following: KAPA Long Range HotStart PCR Kit (Sigma-Aldrich, UK: Nuclease-free H2O for a final volume of 50 μl, 5 μl 3 ng/μl sample DNA [total 15 ng template], 10 μl 5 × buffer solution, 1 μl 1.0 mg/ml BSA, 1 μl 10 mM dNTP mix, 2.5 μl each 10 μM primer, 3.5 μl 25 mMgCl2, 0.5 μl 2.5 U/μl KAPA HotStart polymerase). **Note:** Primer nucleotide sequences and corresponding conditions are outlined in Table 1. These 50 μl mixes included both a no template control, and a 50% control, containing control DNA. These control samples ensured a lack of contamination in the reaction component and to ensure quantitative conditions within the linear range of fragment amplification, respectively.

**Table 1:** Utilised primers (forward and reverse) in human and C2C12 samples. All primers were previously validated in target samples (Furda et al., 2012; Ayala-Torres et al., 2000; Gonzalez-Hunt et al., 2016).

Species	Sequence	Annealing Temp	Size
*Homo Sapiens*	**F:** 5′-TCTAAGCCTCCTTATTCGAGCCGA-3′	64 °C	8.9 kb
	**R:** 5′-TTTCATCATGCGGAGATGTTGGATGG-3′		
*Homo Sapiens*	**F:** 5′-CCCCACAAACCCCATTACTAAACCCA-3′	62 °C	221 bp
	**R:** 5′-TTTCATCATGCGGAGATGTTGGATGG-3′		
*M. Musculus*	**F:** 5′-GCCAGCCTGACCCATAGCCATAATAT-3′	64 °C	10.9 kb
	**R:** 5′-GAGAGATTTTATGGGTGTAATGCGG-3′		
*M. Musculus*	**F:** 5′-CCCAGCTACTACCATCATTCAAGT-3′	60 °C	117
	**R:** 5′-GATGGTTTGGGAGATTGGTTGATGT-3′		

Each sample was vortexed briefly and subsequently centrifuged for approximately 10 s before being aliquoted into the well of a PCR plate at a volume of 25 μl. PCR products were amplified based on the conditions presented in Table 2.

**Table 2:** Thermocycler variables associated with long- and short-mitochondrial primers.

Phase	Long Mito Primers	Short Mito Primers
*Melting*	94 °C for 4 min	94 °C for 1 min
*Amplification*	26-28 cycles of melting (94 °C for 15 s)	20 cycles of melting (94 °C for 15 s)
*Annealing*	66 °C for 12 min	60 °C for 45 s
*Final Extension*	72 °C for 10 min	72 °C for 45 s
*Hold*	4 °C (or 8 °C if being held overnight)	4 °C (or 8 °C if being held overnight)

To quantify products, 90 μl of TE buffer was added to 10 μl of PCR product and performed in triplicated within a 96-well PCR plate. Fluorescence was quantified via PicoGreen incubated in the dark for 10-min, and measured with at an excitation of 485 nm and emission of 530 nm. Each of the triplicate sample values were averaged and subtracted from the no template control and fluorescence from the fluorescence values for the PCR products, including the 50% control.

Large mitochondrial PCR products were subsequently normalised for copy number using fluorescence values of small mitochondrial PCR products as recommended by Furda *et al.* (2012). Before normalising samples, a correction factor was quantified by dividing each of the small mitochondrial PCR products by the mean of all the small mitochondrial products. Subsequently, the normalised fluorescence values were divided into each sample by the average normalised fluorescence value to give the amplification relative to the control. Finally, a negative natural log (-ln) was performed on each amplification figure to quantify the lesion frequency per fragment. This was normalised to the number of lesions/10 kb to quantify the extent of mtDNA damage.

### 2.6 DNA single strand breaks

DNA damage was measured in PBMCs using the single cell gel electrophoresis termed the comet assay [43]. Briefly, 50 μl of isolated lymphocytes were mixed with 150 μl of low melting point agarose, of which 70 μl was layered on to normal agarose slides and allowed to solidify under coverslips at 4 °C. After 5 min, the coverslips were removed and placed in lysis buffer (2.5 M NaCl, 100 mM NaEDTA, 10 mM Trizma, 1% Trition-X, pH 10) for 1 h at 4 °C. Slides were then placed in electrophoresis solution (300 mM NaOH, 1 mM EDTA, pH 12.5–13) for a 20-min incubation period, followed by 30 min electrophoresis at 4 °C (0.83 V/cm). Slides then underwent neutralisation followed by staining using SYBR® Gold. 50 random cells were counted on each slide using an Olympus BH-2 epifluorescence microscope. Slides were prepared and counted in duplicate with the mean DNA damage recorded. All steps were carried out in the dark to prevent further DNA damage. A H_2_O_2_ (Sigma-Aldrich, U.K.) series was performed by incubating lymphocytes for 20 min at different dilutions, to act as a positive control for the standard alkaline comet assay. The intra/inter-assay co-efficient of variation (CV) are <8%.

### 2.7 C2C12 cell culture

Mus Muculus C2C12 myoblasts (American Type Culture Collection, CRL-1772) were incorporated to provide an insight to the mechanisms of oxidative damage to DNA by acting as a positive control. Cell cultures were grown as previously described by Kislinger *et al.* [44] in Dulbecco's Modified Eagle's Medium (Invitrogen) supplemented with 20% fetal bovine serum, 200 mM l-glutamine, 10 units/ml penicillin, and 10 μg/ml streptomycin at 37 °C in a humified atmosphere of 5% CO_2_. Adherent cells were harvested by trypsin incubation using 0.05% trypsin in EDTA (Gibco, USA) and seeded at a density of 1 × 10^5^. Cell viability was assessed using the trypan blue exclusion assay (Sigma-Aldrich), with all experimental cells exceeding ≥95% viability [45]. All samples were incubated with exogenously applied H_2_O_2_ of an incrementally increasing concentration for 30-min. This was performed at 4 °C to attenuate DNA repair.

### 2.8 Lipid hydroperoxides (LOOH)

Serum LOOH were measured spectrophotometrically using the method of Wolff [46]. Briefly, ferrous oxidation of Xylenol Orange (FOX) was used to quantify the oxidation of ferrous (Fe^2+^) iron to ferric (Fe^3+^) iron ions, and the subsequent binding of Fe^3+^ to the FOX-1 reagent. The intra/inter-assay coefficient of variation (CV) is <5%.

### 2.9 Lipid soluble antioxidants (LSA)

LSA were analysed by simultaneous determination using high-performance liquid chromatography (HPLC) as described by Thurnham *et al.* [47]. Serum samples were measured under the same testing parameters outlined in McClean *et al.* [48] for α-tocopherol, γ-tocopherol, retinol, xanthophyll, and β-carotene at changing wavelengths. The intra/inter-assay coefficient of variation (CV) is <7%.

### 2.10 Electron paramagnetic resonance (EPR) spectroscopy

The ascorbyl free radical was quantified using EPR on a Bruker EMX EPR spectrometer (Bruker Instruments Inc., Billerica, MA, USA) as described by Williamson *et al.* [49]. 1 ml plasma was mixed thoroughly with 1 ml dimethyl sulfoxide in a glass test tube, and 1 ml of the final solution was drawn into a sterile syringe and flushed into the analyser cavity. The spectrometer parameter conditions were set as follows: frequency (9.785 GHz); microwave power (20 mW); modulation frequency (100 kHz) and modulation amplitude (1.194 G) for three sweeps. Spectral parameters were obtained using commercially available software (Bruker Win EPR System, Version 3.2) and filtered identically. The relative concentration of the ascorbyl radical was determined by signal intensity. All samples were analysed at room temperature.

### 2.11 Statistical analysis

SPSS statistical software (IBM, Surrey, UK, v.25) was used to analyse data sets, and data normality was determined using the Shapiro-Wilks test (P > 0.05). Data was analysed using a two-way ANOVA with alpha *P* < 0.05. Following a significant interaction effect, between group differences were analysed using a one-way ANOVA, while a Bonferroni paired samples *t*-test was used for within time differences. All data are represented as mean (M) ± standard deviation (SD), with the expection of mtDNA damage, where error bars represent mean ± standard error of the mean as recommended by Hunter *et al.* (2010) and Furda *et al.* (2012). The magnitude of change was expressed as partial eta squared (effect size, ES) throughout.

## 3 Results

### 3.1 Compliance

Assessment of adherence was ascertained through two indirect measures to improve accuracy of reporting: pill/bottle counts and questioning the participant during laboratory visits [50]. All 24 participants (100%) completed both the acute and chronic phases of the study. From a total of 528 administration opportunities (across all participants, and all phases), only 2 tablets were missed, thus compliance was 99.6% for the entire study. No adverse side effects as a result of MitoQ consumption were reported during the supplementation period.

### 3.2 Heart rate

**Fig. 2:**
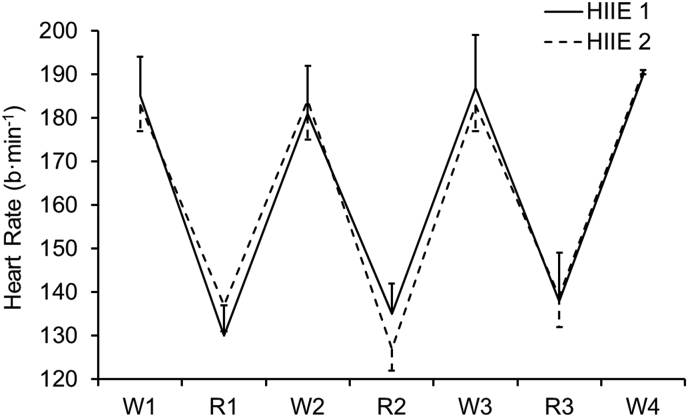
Heart rate (b·min^−1^) across high-intensity intermittent exercise. Note: HIIE 1 refers to exercise completed during the acute phase, whereas HIIE 2 refers to exercise completed during the chronic phase. Heart rate across both trials was similar (P > 0.05). Average workload across work- and rest-intervals was 380 ± 137 W and 156 ± 102 W, respectively.

Heart rate across exercise trials, and following acute and chronic supplementation, were similar (P > 0.05) as shown in Fig. 2.

### 3.3 Lymphocyte nuclear DNA damage

#### 3.3.1 Acute-supplementation

**Fig. 3:**
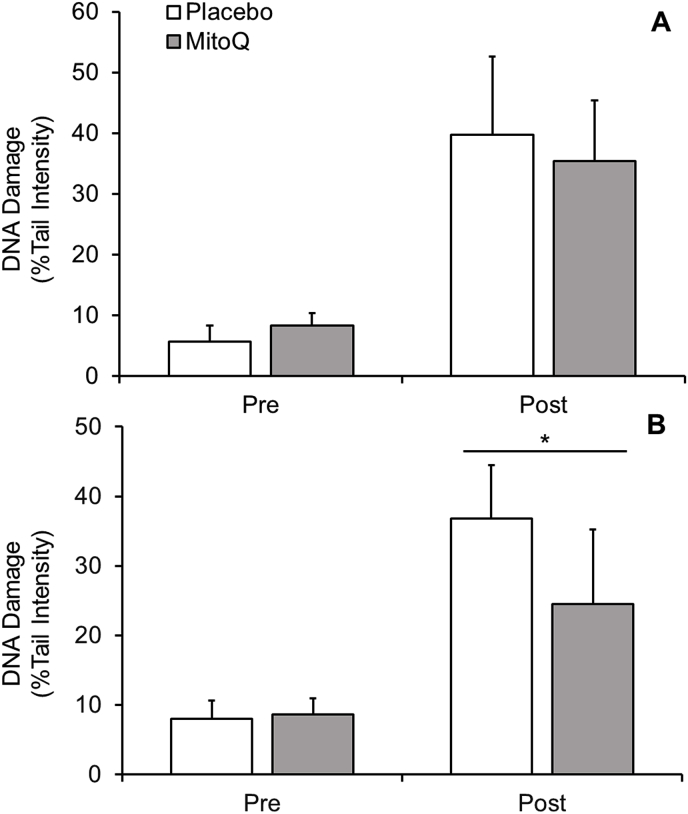
Lymphocyte DNA damage following HIIE in (**A**) acute and (**B**) chronic MitoQ supplementation. Significant within group interaction effect (P < 0.05) was observed (not shown).* denotes a significant interaction effect (P < 0.05) between groups.

Exercise-induced DNA damage following acute MitoQ supplementation is depicted in Fig. 3A. There was an interaction effect for time × group (P < 0.05, ES = 0.4), and post-hoc analysis shows a difference between pre- and post-exercise time points for each intervention group (P < 0.05, ES = 0.7, [Placebo = Δ34.1%; MitoQ = Δ27.1%]). Additionally, there was a main effect for time (pooled group pre-vs. post-exercise, P < 0.05, ES = 0.7). No interaction effect between groups was observed (P > 0.05).

#### 3.3.2 Chronic supplementation

Following chronic MitoQ supplementation (Fig. 3B), there was a time × group interaction effect (P < 0.05, ES = 0.5), with post-hoc analysis demonstrating a difference between placebo and MitoQ groups post-exercise (P < 0.05, ES = 0.2). Furthermore, there was a within (pre-to post-exercise) group effect (P < 0.05, ES = 0.4, [Placebo = Δ28.9%; MitoQ = Δ15.9%]).

### 3.4 Lymphocyte mitochondrial DNA damage

#### 3.4.1 Acute-supplementation

**Fig. 4:**
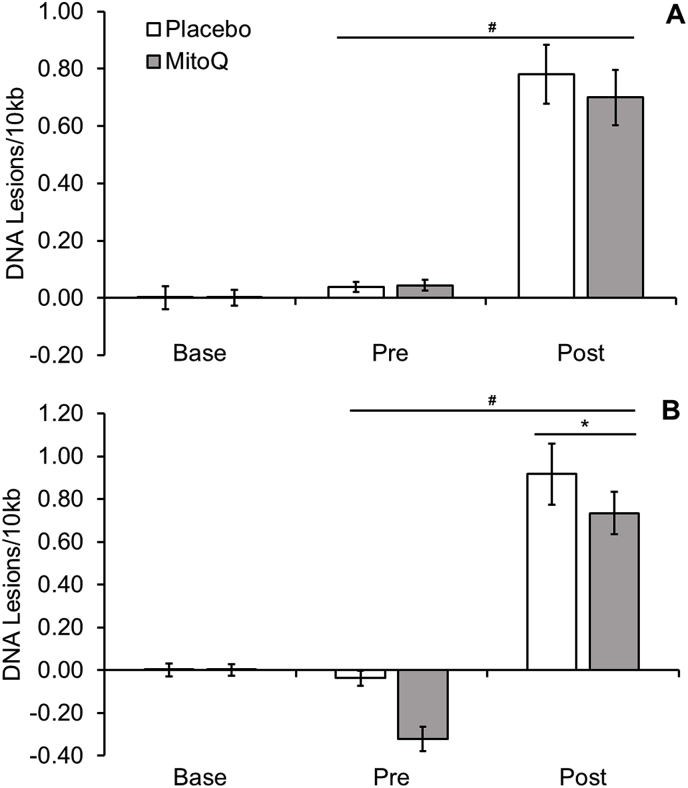
Lymphocyte mitochondrial DNA damage following HIIE in (A) acute and (B) chronic MitoQ supplementation. Note: # indicates a main effect of time (P < 0.05). * represents a significant interaction effect of time x group (P < 0.05).

An increase in mtDNA damage (P < 0.05, ES = 0.53, see Fig. 4A) was observed following HIIE. There was no difference in mtDNA damage between baseline and pre-exercise time points after supplementation (P > 0.05). No between group differences were observed between pre- and post-exercise time points (P > 0.05), suggesting acute supplementation of MitoQ failed to protect against exercise induced mtDNA damage.

#### 3.4.2 Chronic supplementation

There was an increase in mtDNA damage following exercise (pooled data; P < 0.05, ES = 0.53, see Fig. 4B). An interaction effect of time and group was also observed (P < 0.05, ES = 0.29), consistent with MitoQ protecting against exercise-induced mtDNA damage.

### 3.5 Human muscle mitochondrial DNA damage

#### 3.5.1 Chronic supplementation

**Fig. 5:**
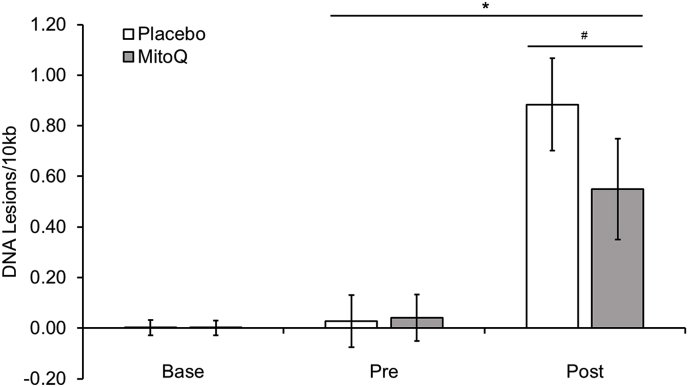
Muscle mitochondrial DNA damage following HIIE and chronic MitoQ supplementation. Note: ^#^ indicates a main effect of time (P < 0.05)., and * represents a significant interaction effect of time x group (P < 0.05).

There was a time × group interaction effect at post-exercise (P < 0.05, ES = 0.38, see Fig. 5), indicating that chronic MitoQ supplementation protects against mtDNA damage in muscle.

### 3.6 Mouse C2C12 mitochondrial DNA damage

**Fig. 6:**
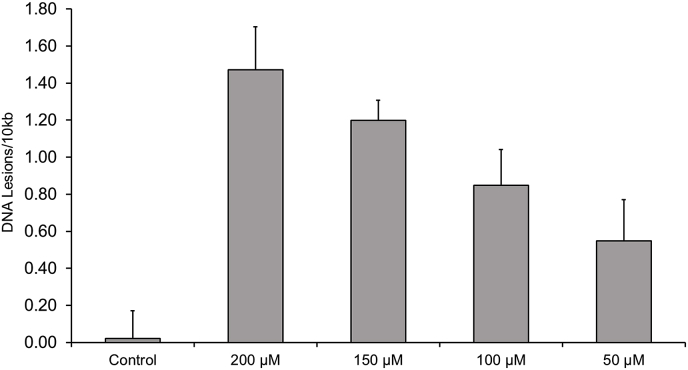
Hydrogen peroxide series on C2C12 mouse muscle cells; acting as positive control for LA-qPCR utilised in human muscle.

Mitochondrial DNA damage in C2C12 myoblast cells demonstrated a dose-dependent increase following exposure to H_2_O_2_ (Fig. 6). The observed damage at 50–100 μM is similar to that observed in our human lymphocyte and muscle cells.

### 3.7 Lipid hydroperoxides

#### 3.7.1 Acute supplementation

**Fig. 7:**
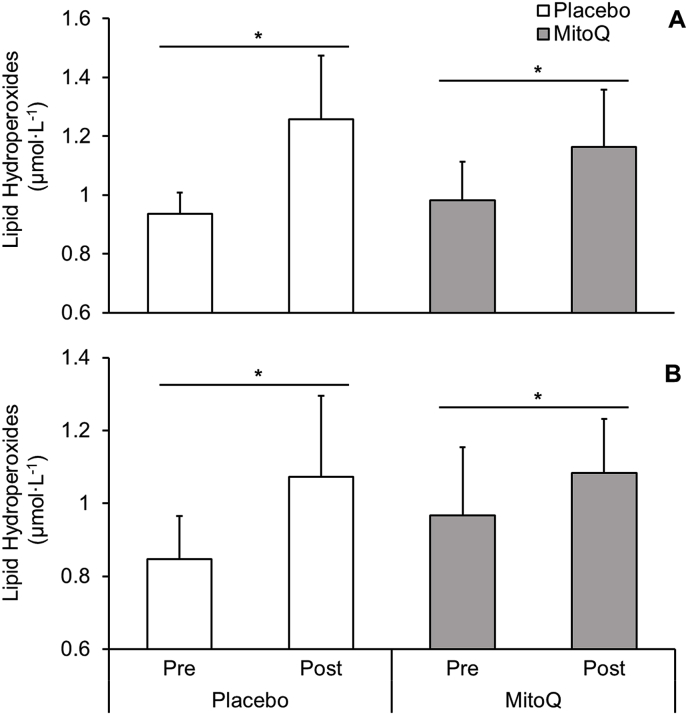
(**A**) Lipid hydroperoxides following HIIE across the acute phase. (**B**) Lipid hydroperoxides following HIIE across the chronic phase. * represents a significant within group effect (P < 0.05).

There was no time × group interaction effect for LOOH's following acute MitoQ treatment (P > 0.05, ES = 0.14, Fig. 7A). However, lipid hydroperoxides increased in placebo (Δ34%, P < 0.005) and MitoQ groups (Δ19%, P = 0.029) post-exercise.

#### 3.7.2 Chronic supplementation

There was no between group effect (P > 0.05, ES = 0.21) as observed in Fig. 7B. However, a within group effect in both the placebo (Δ27%, P = 0.03) and MitoQ (Δ12%, P = 0.01) groups was observed.

### 3.8 Ascorbyl free radical

There was no time × group interaction effect in ascorbyl free radical concentration (*P* > 0.05) following acute or chronic supplementation (Table 3). However, further analysis of pooled data (pre-vs. post-exercise) shows a main effect of time in both acute (*P* = 0.02) and chronic (*P* = 0.03) experimental trials.

**Table 3:** Ascorbyl free radical concentration in blood plasma following exercise in acute and chronic supplementation phases. † denotes a main effect of time (pooled pre- and post-exercise, P < 0.05). au = arbitrary units.

	Acute			Chronic		
	Pre- Ex	Post-Ex	Δ%	Pre- Ex	Post-Ex	Δ%
MitoQ (au)	170 ± 167	273 ± 144^†^	60.6	183 ± 159	303 ± 159^†^	65.6
Placebo (au)	189 ± 120	257 ± 127^†^	35.9	168 ± 141	299 ± 203^†^	77.9

### 3.9 Lipid soluble antioxidants

There was no interaction effect across any LSA parameter (*P* > 0.05, see Table 4), however, there was a main effect for time in *α-*tocopherol for both acute (pooled data; *P* < 0.05, ES = 0.1) and chronic (pooled data; *P* < 0.05, ES = 0.2) experimental phases.

**Table 4:** Serum lipid soluble antioxidant concentration following exercise in acute and chronic supplementation phases. All values are expressed as mmol·L^−1^. ^†^ denotes a main effect of time (pooled data; p < 0.05).

	Acute		Chronic	
	Pre-Ex	Post-Ex	Pre-Ex	Post-Ex
*α-Tocopherol*				
MitoQ	19.82 ± 0.9	22.97 ± 4.3^†^	19.21 ± 0.6	25.69 ± 4.6^†^
Placebo	18.13 ± 1.7	22.71 ± 3.5^†^	17.62 ± 1.2	22.27 ± 3.2^†^
*γ-Tocopherol*				
MitoQ	1.53 ± 0.4	1.41 ± 0.3	1.63 ± 0.3	1.81 ± 0.4
Placebo	1.72 ± 0.2	1.83 ± 0.7	1.81 ± 0.6	1.93 ± 0.8
*β-Carotene*				
MitoQ	0.27 ± 0.08	0.29 ± 0.06	0.23 ± 0.07	0.24 ± 0.09
Placebo	0.26 ± 0.07	0.24 ± 0.08	0.27 ± 0.07	0.24 ± 0.11
*Xanthophyll*				
MitoQ	0.20 ± 0.09	0.25 ± 0.07	0.23 ± 0.11	0.26 ± 0.07
Placebo	0.18 ± 0.11	0.21 ± 0.09	0.21 ± 0.12	0.25 ± 0.09
*Retinol*				
MitoQ	1.83 ± 0.4	1.89 ± 0.6	1.69 ± 0.4	1.90 ± 0.8
Placebo	1.76 ± 0.6	1.71 ± 0.9	1.88 ± 0.4	2.13 ± 0.6

## 4 Discussion

To date, knowledge of how exercise impacts mtDNA damage is lacking [7,9]. Recently, it has been proposed that the simultaneous, beneficial (e.g., signal) and harming (e.g., damage to macromolecules) effects of exercise induce a state of oxidative eustress, which is largely dependent on the local microenvironment (pH, temperature, solvent accessibility, and vicinal interactome) [51]. Building on the limited body of literature, we offer novel insights into the role of exercise-induced redox perturbations in mitochondria. We demonstrate an increase in DNA damage (mitochondrial and nuclear) and lipid peroxidation, in tandem with the detection of the ascorbyl free radical suggesting that HIIE increases the generation of reactive species [52]. Similarly, the presence of the ascorbyl free radical suggests that ascorbic acid is oxidised to potentially scavenge other free radicals including, O_2_·-, hydroxyl, and lipid-derived alkoxyl and peroxyl radicals [53,54]. Together the increase in oxidative damage within PBMC (nuclear and mtDNA), muscle tissue (mtDNA), and corresponding changes to lipid hydroperoxides, lipid soluble antioxidants, and the presence of the ascorbyl free radical signifies perturbed redox homeostasis.

One major finding is that exercise increased DNA damage in nucleus and mitochondria. While several factors (e.g., lack of histones) render mtDNA more susceptible to damage than nuclear DNA, the chemistry underlying the observed damage is similar (reviewed in Refs. [55]). In brief, local H_2_O_2_ reacts with accessible transition metals to produce the damaging hydroxyl radical species via Fenton-type chemistry (H_2_O_2_ + Fe^2+^ → Fe^3+^ + –OH + ·OH [*k* ~ 76 M^−1^ s^−1^]; H_2_O_2_ + Cu^+^ → Cu^2+^ + –OH + ·OH [*k* ~ 4.7 × 10^3^ M^−1^ s^−1^]) [55,56]. Note, the rate for Fe^2+^ increases by 2–3 orders of magnitude when it is bound to citrate or ATP. Hydroxyl radical reacts with DNA bases at diffusion-controlled rates (*k* ~ 5–8 × 10^9^ M^−1^ s^−1^ for guanine [57]); potentially generating other end products which can further propagate oxidative damage [58]. It is also worth highlighting exercise-induced peroxynitrite-derived radicals, namely carbonate and nitrogen dioxide radicals, could underlie some of the observed DNA damage [59]. Mechanistically, exercise could increase DNA damage by: (1) inhibiting repair; (2) increasing H_2_O_2_ production; and/or (3) increasing the labile redox-active transition metal pool. It follows that, one or more factors must simultaneously operate in the nucleus and mitochondria during exercise. The observed nuclear DNA damage likely requires H_2_O_2_ diffusion and/or generation proximally to the nuclear genome [55]. Although this likely occurs from other cellular organelles (such as the endoplasmic reticulum, or membrane-bound NADPH oxidases), the mitochondria may also contribute to nuclear DNA damage. Murphy (2012)[60] outlines that mitochondrial H_2_O_2_ has a large capacity for diffusion, and is hypothesised to play distinct roles in downstream physiological signalling responses, including post-translational modifications [61,62]. This retrograde signalling from the mitochondria to the nuclear domain may account for the potential of mitochondrial H_2_O_2_ to instigate nuclear DNA damage [55]. Given the topical interest regarding 2 above, and combined with the ability of our findings to reignite debate concerning the source of exercise-induced superoxide, we make three points:

1. Mitochondrial superoxide production. Exercise is understood to reduce mitochondrial net O_2_·- production by increasing ATP demand. The continued availability of NADH means the flavin mononucleotide (FMN) group of complex I continues to produce superoxide at an appreciable rate in metabolic conditions mimicking exercise in isolated mitochondria. A potential source of the necessary H_2_O_2_, therefore, continues to operate. Moreover, the vast majority of redox research in an exercise setting recapitulates linear moderate/high intensity work and it could be argued the active recovery employed within this study creates a switch in ATP demand, potentially increasing mitochondrial O_2_·-/H_2_O_2_ production [63]; this highlights our limited understanding of exercising human *in vivo* mitochondrial redox dynamics. That is, cyclic fluxes in work done may induce oscillatory mitochondrial superoxide behaviour (i.e., troughs during the bout and peaks during the rest). Further, the persistent nature of mitochondrial superoxide production means a low rate of net production can cause damage over time.
2. Mitochondrial antioxidant defence. In the absence of an overt increase in superoxide production, several factors could decrease mitochondrial antioxidant defence by limiting the activity of the NADPH dependent glutathione and thioredoxin systems. For example, ADP demand induce depolarisation may limit the activity of the proton motive force consuming transhydrogenase to decrease NADPH dependent defence.
3. Cytosolic superoxide production. The present work details the mtDNA damage response in C2C12 myoblasts to H_2_O_2_ incubation at various concentrations from 50 to 200 μM by sequestering the high concentrations of extramitochondrial H_2_O_2_ and recapitulating the exercise-induced Fenton reactions with transition metal ions; this dose-dependent response is indicative of other investigations using similar models [[64], [65], [66]]. The biological relevance of this is unclear, and future work should include a propidium iodine exclusion assay to ascertain the mtDNA damage threshold by which H_2_O_2_ incubation induces cell death. In support, Goncalves et al. (2015, 2020) observed the greatest rate of H_2_O_2_ production occurs under resting conditions, and becomes incrementally lower as exercise intensity increases as the redox centres that donate electrons to O_2_ become more oxidised. It could be hypothesised that as exercise intensity increases, H_2_O_2_ production from the electron transport chain decreases while simultaneously increasing the ability of the mitochondria to sequester extramitochondrial H_2_O_2_; thereby increasing the likelihood of Fenton-mediated reactions, and subsequent mtDNA damage. While the dose of H_2_O_2_ used is non-physiological, it could be that H_2_O_2_ diffusing in, increases mtDNA damage.

We show, for the first time, that MitoQ decreases exercise-induced DNA damage. We interpret the ability of MitoQ to decrease exercise-induced DNA damage as beneficial. While we acknowledge oxidative damage adducts can signal, an increase in mtDNA damage is likely to be harmful. That said, it would be intriguing to determine whether an increase in mtDNA damage and the associated sensing is linked to mitochondrial biogenesis (i.e., repair could be coupled to an adaptive redox regulated response). If so, it is conceivable that MitoQ may be disadvantageous. Mechanistically, it is plausible that MitoQ offers protection to complexes I and IV (cytochrome c oxidase) of the electron transport chain from direct oxidative damage [[68], [69], [70], [71]]. MitoQ may also indirectly affect superoxide production via reverse electron transfer by interacting with protonmotive forces (uptake decreases proton motive force) and the coenzyme Q pool redox state as detailed by Robb *et al.* [72]. It could also attenuate complex III mediated superoxide production by dissipating the proton motive force. Secondly, the prophylactic effects of MitoQ supplementation in PBMCs and skeletal muscle tissue observed could be attributable to direct scavenging of free radicals (RO· + MitoQH_2_ → ROH + MitoQ·^-^). If superoxide was protonated (as is postulated to occur at complex III [73]), then MitoQ could intercept a diffusing species directly. Although kinetically feasible, it is improbable that MitoQ directly reacts with the hydroxyl radical as this would require MitoQ residing in close vicinity to mtDNA and outcompeting other hydroxyl radical targets. The more probable explanation is the attenuation of a peroxide-derived species as evident by Pham et al. (2020). Further, the mitochondria contain a high prevalence of lipids vulnerable to peroxidation (such as anionic cardiolipin, phosphatidylethanolamine, and phosphatidylcholine; [74]. Lipid peroxidation is generally initiated when a sufficiently reactive (i.e., thermodynamically and kinetically competent) free radical (hydroxyl radical) abstracts a bis-allylic hydrogen atom from a methylene group [75]; consequently, this process is propagated by unstable, adjacent lipid radicals (alkoxyl and peroxyl) [ROOH + Fe^2+^ → ROO^.^ + Fe^3+^ + H^+^] [76,77]. This mechanism is consistent with others who demonstrate a positive correlation between lipid-derived alkoxyl free radicals, and oxidation of DNA and lipids following exercise [53,78]. Although alkoxyl free radicals were not measured in the present study, the increase in lipid peroxidation and DNA damage defines a plausible mechanism. Additionally, lipid-derived radicals formed within the mitochondrial bilayer could be released into a cytosolic environment [79]. It is highly likely that the reduction in mtDNA damage following chronic MitoQ supplementation was caused by the (indirect) attenuation of H_2_O_2_ concentration [34] and lipid-derived oxidants such as peroxyl and alkoxyl species. On a final note, MitoQ supplementation may also offer other prophylactic effects including the upregulation of antioxidant genes (HO-1, NQO-1, γ-GCLC, CAT, GPx [34,70]), and exerting extramitochondrial prophylaxis as it cycles in and out of mitochondria in accordance with the membrane potential.

The inability of MitoQ to affect an acute change is likely attributable to insufficient uptake. There is a scarcity of human studies (especially in healthy, exercising populations) on the appropriate dosage to induce a prophylactic effect, thus, extrapolation of a human recommendation from animal studies is difficult. In addition, the lack of prophylaxis following acute supplementation may be explained by the oral consumption and first pass metabolism of the stomach and liver [80]. As a result, the rate and amount of MitoQ reaching systemic circulation within the hour absorption phase may be limited. Although bioavailability and tolerance of MitoQ has been clarified *in vivo* [22], an appropriate dose and delivery to the site of action have yet to be fully elucidated in human mitochondria. Consistent with increased bioavailability, chronic MitoQ supplementation provided a protective effect to nuclear and mtDNA in PBMCs and human muscle (mtDNA only) following HIIE.

One intriguing insight uncovered was the lack of effect on the pre-exercise DNA damage following chronic MitoQ supplementation. Although this may appear trivial, it highlights our very limited understanding of exercise-mediated modifications to mitochondrial redox dynamics. For one, the majority of emerging evidence would suggest mitochondria generate the greatest amount of O_2_·-/H_2_O_2_ at rest [6,67,81], and as a result, it could be hypothesised that chronic MitoQ supplementation would have altered quiescent mitochondrial and/or nuclear DNA damage. It could potentially be the case that MitoQ does indeed alter State 4 mitochondrial O_2_·-/H_2_O_2_ dynamics which may not be detectable by measures of oxidative damage to DNA or lipids employed within the current study; especially considering the low levels of basal oxidative damage presented in healthy individuals [9,82,83]. This highlights the potential that MitoQ may be predominately applicable in conditions characterised by chronic oxidative stress and/or mitochondrial dysfunction such as chronic kidney disease, cardiovascular disease, chronic obstructive pulmonary disorder, acyl-CoA dehydrogenase deficiency, and neurodegenerative diseases [[84], [85], [86], [87]].

Interestingly, the present study failed to detect any effect of MitoQ supplementation on lipid peroxidation. Although the efficacy of general antioxidant supplementation and exercise-induced oxidative stress has been well characterised [[88], [89], [90], [91]], there is a scarcity of literature surrounding mitochondrial-targeted antioxidants in exercise. Despite different methods employed for the quantification of lipid peroxidation, neither Shill et al. (2016) nor the results of the present study demonstrated a prophylactic effect of chronic MitoQ supplementation. Mechanistically, there are several physiological variables which may explain these findings. The comparatively hydrophilic nature of MitoQ allows it to bind to the matrix-facing surface of the inner-mitochondrial membrane where it is continually recycled to the active antioxidant ubiquinol by complex II [92]. An additional point concerns the ability of coenzyme Q10 to recycle the α-tocopherol radical in the plasma membrane thereby attenuating ferroptosis by metabolising lipid radicals to hydroperoxides (ROO^.^ [or RO^.^]+ α-TOH → ROOH [ROH] + α-TOH·; CoQH_2_ + α-TOH· → α-TOH + CoQ·^-^ radical) [93]; this mechanism could partially account for the lack of variation in lipid hydroperoxides and the reduction in mtDNA damage following chronic MitoQ supplementation. Conversely, our group has previously shown that lipid-derived radicals are released extracellularly [52,78,94]; as such, MitoQ may be unable to interact with these species, which may reconcile the lack of effect of supplementation on systemic lipid peroxidation. Further, the current study quantified lipid hydroperoxides in serum; thus, lacking the sensitivity and/or specificity to distinguish between mitochondrial and non-mitochondrial sources of lipid peroxidation. Future research should consider incorporating several biomarkers (such as F_2_-Isoprostanes, 4-hydroxy-2-trans-nonenal etc.) across multiple tissue types to ascertain the extent, locality, and downstream consequences of targeted antioxidants on lipid peroxidation [[95], [96], [97]]. Moreover, it should be noted that nuclear DNA damage was detected using the comet assay which detects single-strand breaks and alkali-labile sites; however, greater sensitivity for the detection of more specific DNA lesions can be achieved by inclusion of endonuclease III, formamidopyrimidine DNA glycosylase, 8-oxoGua DNA glycosylase, and fluorescent *in situ* hybridisation, as previously applied [[43], [49], [99]].

## 5 Conclusions

Our work demonstrates that HIIE damages mtDNA both systemically in lymphocytes and locally in muscle tissue, occurring in parallel with nuclear DNA damage. While insufficient bioavailability likely explains the inability of acute MitoQ to impact DNA damage in mitochondria or the nucleus, chronic MitoQ supplementation safeguards both genomes against DNA damage in exercising humans. This study adds a number of key concepts to the exercise redox field: (1) HIIE induces mtDNA damage in lymphocytes and human muscle tissue likely through the generation of reactive oxygen and/or lipid-derived species; (2) Chronic MitoQ supplementation offers a prophylactic effect possibly by (i) directly or indirectly metabolising key reactive species, (ii) altering the activities of respiratory chain complexes, and/or (iii) exerting extramitochondrial effects; and (3) mitochondria may act as a sink to cytosolic H_2_O_2_. However, the relevance of this in exercising human muscle is unknown. This study offers novel, mechanistic insights to mitochondrial redox dynamics and targeted supplementation, and presents a number of exciting avenues for future research (e.g., it is unknown if MitoQ may impair or enhance exercise adaptations that are dependent on mitochondrial metabolism and dynamics). Lastly, the notion that a protective effect of a mitochondria-targeted antioxidant was only unmasked by exercise, reinforces the value of interrogating multiple physiological states when appraising the efficacy of an antioxidant.

## Source of funding

This research did not receive any specific grant from funding agencies in the public, commercial, or not-for-profit sectors.

## Declaration of competing interest

No potential conflict of interest was reported by the authors.

## References

[1] Murphy M.P. (2016). Understanding and preventing mitochondrial oxidative damage. Biochem. Soc. Trans..

[2] Chakrabarty S., Kabekkodu S.P., Singh R.P., Thangaraj K., Singh K.K., Satyamoorthy K. (2018). Mitochondria in health and disease. Mitochondrion.

[3] Sharma P., Sampath H. (2019). Mitochondrial DNA integrity: role in health and disease. Cells.

[4] Singh G., Pachouri U.C., Khaidem D.C., Kundu A., Chopra C., Singh P. (2015). Mitochondrial DNA damage and diseases. F1000Research..

[5] Van Houten B., Hunter S.E., Meyer J.N. (2016). Mitochondrial DNA damage induced autophagy, cell death, and disease. Front. Biosci. - Landmark..

[6] Goncalves R.L.S., Watson M.A., Wong H.S., Orr A.L., Brand M.D. (2020). The use of site-specific suppressors to measure the relative contributions of different mitochondrial sites to skeletal muscle superoxide and hydrogen peroxide production. Redox Biol.

[7] Fogarty M.C., Devito G., Hughes C.M., Burke G., Brown J.C., McEneny J., Brown D., McClean C., Davison G.W. (2013). Effects of α-lipoic acid on mtDNA damage after isolated muscle contractions. Med. Sci. Sports Exerc..

[8] Kino K., Hirao-Suzuki M., Morikawa M., Sakaga A., Miyazawa H. (2017). Generation, repair and replication of guanine oxidation products. Gene Environ..

[9] Tryfidou D.V., McClean C., Nikolaidis M.G., Davison G.W. (2020). DNA damage following acute aerobic exercise: a systematic review and meta-analysis. Sports Med..

[10] Dizdaroglu M., Rao G., Halliwell B., Gajewski E. (1991). Damage to the DNA bases in mammalian chromatin by hydrogen peroxide in the presence of ferric and cupric ions. Arch. Biochem. Biophys..

[11] Ljungman M., Hanawalt P.C. (1992). Efficient protection against oxidative DNA damage in chromatin. Mol. Carcinog..

[12] Richter C., Park J.W., Ames B.N. (1988). Normal oxidative damage to mitochondrial and nuclear DNA is extensive. Proc. Natl. Acad. Sci. U.S.A..

[13] Cline S.D. (2012). Mitochondrial DNA damage and its consequences for mitochondrial gene expression. Biochim. Biophys. Acta - Gene Regul. Mech..

[14] Cobley J.N., Sakellariou G.K., Murray S., Waldron S., Gregson W., Burniston J.G., Morton J.P., Iwanejko L.A., Close G.L. (2013). Lifelong endurance training attenuates age-related genotoxic stress in human skeletal muscle. Longev. Heal..

[15] Mao G., a Kraus G., Kim I., Spurlock M.E., Bailey T.B., Zhang Q., Beitz D.C. (2010). A mitochondria-targeted vitamin E derivative decreases hepatic oxidative stress and inhibits fat deposition in mice. J. Nutr..

[16] Krishna C.M., Liebmann J.E., Kaufman D., DeGraff W., Hahn S.M., McMurry T., Mitchell J.B., Russo A. (1992). The catecholic metal sequestering agent 1,2-dihydroxybenzene-3, 5-disulfonate confers protection against oxidative cell damage. Arch. Biochem. Biophys..

[17] Silveira L.R., Pereira-Da-Silva L., Juel C., Hellsten Y. (2003). Formation of hydrogen peroxide and nitric oxide in rat skeletal muscle cells during contractions. Free Radic. Biol. Med..

[18] Fang Y., Hu X.H., Jia Z.G., Xu M.H., Guo Z.Y., Gao F.H. (2012). Tiron protects against UVB-induced senescence-like characteristics in human dermal fibroblasts by the inhibition of superoxide anion production and glutathione depletion. Australas. J. Dermatol..

[19] Finichiu P.G., Larsen D.S., Evans C., Larsen L., Bright T.P., Robb E.L., Trnka J., Prime T.A., James A.M., Smith R.A.J., Murphy M.P. (2015). A mitochondria-targeted derivative of ascorbate: MitoC. Free Radic. Biol. Med..

[20] Murphy M.P., Hartley R.C. (2018). Mitochondria as a therapeutic target for common pathologies. Nat. Rev. Drug Discov..

[21] Sohal R.S., Kamzalov S., Sumien N., Ferguson M., Rebrin I., Heinrich K.R., Forster M.J. (2006). Effect of coenzyme Q10 intake on endogenous coenzyme Q content, mitochondrial electron transport chain, antioxidative defenses, and life span of mice. Free Radic. Biol. Med..

[22] Smith R.A.J., Murphy M.P. (2010). Animal and human studies with the mitochondria-targeted antioxidant MitoQ. Ann. N. Y. Acad. Sci..

[23] Ross M.F., Kelso G.F., Blaikie F.H., James A.M., Cocheme H.M., Filipovska A., Ros T.D., Hurd T.R., Smith R.A.J., Murphy M.P. (2005). Lipophilic triphenylphosphonium cations as tools in mitochondrial bioenergetics and free radical biology. Biokhimiya.

[24] Asin-Cayuela J., Manas A.R.B., James A.M., Smith R.A.J., Murphy M.P. (2004). Fine-tuning the hydrophobicity of a mitochondria-targeted antioxidant. FEBS Lett..

[25] James A.M., Sharpley M.S., Manas A.R.B., Frerman F.E., Hirst J., Smith R.A.J., Murphy M.P. (2007). Interaction of the mitochondria-targeted antioxidant MitoQ with phospholipid bilayers and ubiquinone oxidoreductases. J. Biol. Chem..

[26] Kelso G.F., Porteous C.M., Coulter C.V., Hughes G., Porteous W.K., Ledgerwood E.C., Smith R.A.J., Murphy M.P. (2001). Selective targeting of a redox-active ubiquinone to mitochondria within cells: antioxidant and antiapoptotic properties. J. Biol. Chem..

[27] Neuzil J., Widén C., Gellert N., Swettenham E., Zobalova R., Dong L.F., Wang X.F., Lidebjer C., Dalen H., Headrick J.P., Witting P.K. (2007). Mitochondria transmit apoptosis signalling in cardiomyocyte-like cells and isolated hearts exposed to experimental ischemia-reperfusion injury. Redox Rep..

[28] Esplugues J.V., Rocha M., Nuñez C., Bosca I., Ibiza S., Herance J.R., Ortega A., Serrador J.M., D'Ocon P., Victor V.M. (2006). Complex I dysfunction and tolerance to nitroglycerin: an approach based on mitochondrial-targeted antioxidants. Circ. Res..

[29] Graham D., Huynh N.N., Hamilton C.A., Beattie E., Smith R.A.J., Cochemé H.M., Murphy M.P., Dominiczak A.F. (2009). Mitochondria-targeted antioxidant mitoq10 improves endothelial function and attenuates cardiac hypertrophy. Hypertension.

[30] Supinski G.S., Murphy M.P., Callahan L.A. (2009). MitoQ administration prevents endotoxin-induced cardiac dysfunction. Am. J. Physiol. Regul. Integr. Comp. Physiol..

[31] Lowes D.A., Thottakam B.M.V., Webster N.R., Murphy M.P., Galley H.F. (2008). The mitochondria-targeted antioxidant MitoQ protects against organ damage in a lipopolysaccharide-peptidoglycan model of sepsis. Free Radic. Biol. Med..

[32] Margaritelis N.V., Cobley J.N., Paschalis V., Veskoukis A.S., Theodorou A.A., Kyparos A., Nikolaidis M.G. (2016). Principles for integrating reactive species into in vivo biological processes: examples from exercise physiology. Cell. Signal..

[33] Cobley J.N. (2020). How Exercise Induces Oxidative Eustress.

[34] Pham T., MacRae C.L., Broome S.C., D’souza R.F., Narang R., Wang H.W., Mori T.A., Hickey A.J.R., Mitchell C.J., Merry T.L. (2020). MitoQ and CoQ10 supplementation mildly suppresses skeletal muscle mitochondrial hydrogen peroxide levels without impacting mitochondrial function in middle-aged men. Eur. J. Appl. Physiol..

[35] Shill D.D., Southern W.M., Willingham T.B., Lansford K.A., McCully K.K., Jenkins N.T. (2016). Mitochondria-specific antioxidant supplementation does not influence endurance exercise training-induced adaptations in circulating angiogenic cells, skeletal muscle oxidative capacity, or maximal oxygen uptake. J. Physiol..

[36] Rossman M.J., Santos-Parker J.R., Steward C.A.C., Bispham N.Z., Cuevas L.M., Rosenberg H.L., Woodward K.A., Chonchol M., Gioscia-Ryan R.A., Murphy M.P., Seals D.R. (2018). Chronic supplementation with a mitochondrial antioxidant (MitoQ) improves vascular function in healthy older adults. Hypertension.

[37] Jamnick N.A., Botella J., Pyne D.B., Bishop D.J. (2018). Manipulating graded exercise test variables affects the validity of the lactate threshold and V_ O2peak. PloS One.

[38] Helgerud J., Høydal K., Wang E., Karlsen T., Berg P., Bjerkaas M., Simonsen T., Helgesen C., Hjorth N., Bach R., Hoff J. (2007). Aerobic high-intensity intervals improve V̇O2max more than moderate training. Med. Sci. Sports Exerc..

[39] Dill D.B., Costill D.L. (1974). Calculation of percentage changes in volumes of blood, plasma, and red cells in dehydration. J. Appl. Physiol..

[40] Dacie S.M., Lewis J.V. (1968). Practical Haematology.

[41] Hunter S.E., Jung D., Di Giulio R.T., Meyer J.N. (2010). The QPCR assay for analysis of mitochondrial DNA damage, repair, and relative copy number. Methods.

[42] Furda A.M., Bess A.S., Meyer J.N., Van Houten (2012). Analysis of DNA damage and repair in nuclear and mitochondrial DNA of animal cells using quantitative PCR. Methods Mol. Biol..

[43] Davison G.W. (2016). Exercise and oxidative damage in nucleoid DNA quantified using single cell gel electrophoresis: present and future application. Front. Physiol..

[44] Kislinger T., Gramolini A.O., Pan Y., Rahman K., MacLennan D.H., Emili A. (2005). Proteome dynamics during C2C12 myoblast differentiation. Mol. Cell. Proteomics.

[45] Sidlauskaite E., Gibson J.W., Megson I.L., Whitfield P.D., Tovmasyan A., Batinic-Haberle I., Murphy M.P., Moult P.R., Cobley J.N. (2018). Mitochondrial ROS cause motor deficits induced by synaptic inactivity: implications for synapse pruning. Redox Biol.

[46] Wolff S.P. (1994). Ferrous ion oxidation in presence of ferric ion indicator Xylenol Orange for measurement of hydroperoxides. Methods Enzymol..

[47] Thurnham D.I., Smith E., Flora P.S. (1988). Concurrent liquid-chromatographic assay of retinol, alpha-tocopherol, beta-carotene, alpha-carotene, lycopene, and beta-cryptoxanthin in plasma, with tocopherol acetate as internal standard. Clin. Chem..

[48] McClean C., Harris R.A., Brown M., Brown J.C., Davison G.W. (2015). Effects of exercise intensity on postexercise endothelial function and oxidative stress. Oxid. Med. Cell. Longev..

[49] Williamson J., Hughes C.M., Burke G., Davison G.W. (2020). A combined γ-H2AX and 53BP1 approach to determine the DNA damage-repair response to exercise in hypoxia. Free Radic. Biol. Med..

[50] Matsui D. (2009). Strategies to measure and improve patient adherence in clinical trials. Pharmaceut. Med..

[51] Booth D.M., Enyedi B., Geiszt M., Várnai P., Hajnóczky G. (2016). Redox nanodomains are induced by and control calcium signaling at the ER-mitochondrial interface. Mol. Cell..

[52] Davison G.W., Morgan R.M., Hiscock N., Garcia J.M., Grace F., Boisseau N., Davies B., Castell L., McEneny J., Young I.S., Hullin D., Ashton T., Bailey D.M. (2006). Manipulation of systemic oxygen flux by acute exercise and normobaric hypoxia: implications for reactive oxygen species generation. Clin. Sci..

[53] Davison G.W., Ashton T., George L., Young I.S., McEneny J., Davies B., Jackson S.K., Peters J.R., Bailey D.M. (2008). Molecular detection of exercise-induced free radicals following ascorbate prophylaxis in type 1 diabetes mellitus: a randomised controlled trial. Diabetologia.

[54] Piloni N.E., Puntarulo S. (2016). A simple kinetic model to estimate ascorbyl radical steady state concentration in rat central nervous System. Effect of Subchronic Fe Overload, Bioenerg. Open Access.

[55] Cobley J.N., Margaritelis N.V., Morton J.P., Close G.L., Nikolaidis M.G., Malone J.K. (2015). The basic chemistry of exercise-induced DNA oxidation: oxidative damage, redox signaling, and their interplay. Front. Physiol..

[56] Close G.L., Ashton T., McArdle A., Jackson M.J. (2005). Microdialysis studies of extracellular reactive oxygen species in skeletal muscle: factors influencing the reduction of cytochrome c and hydroxylation of salicylate. Free Radic. Biol. Med..

[57] Chatgilialoglu C., D'Angelantonio M., Kciuk G., Bobrowski K. (2011). New insights into the reaction paths of hydroxyl radicals with 2′-deoxyguanosine. Chem. Res. Toxicol..

[58] Dizdaroglu M., Jaruga P. (2012). Mechanisms of free radical-induced damage to DNA. Free Radic. Res..

[59] Cobley J.N., Close G.L., Bailey D.M., Davison G.W. (2017). Exercise redox biochemistry: conceptual, methodological and technical recommendations. Redox Biol.

[60] Murphy M.P. (2012). Mitochondrial thiols in antioxidant protection and redox signaling: distinct roles for glutathionylation and other thiol modifications. Antioxidants Redox Signal..

[61] Finkel T. (2012). Signal transduction by mitochondrial oxidants. J. Biol. Chem..

[62] Go Y.M., Chandler J.D., Jones D.P. (2015). The cysteine proteome. Free Radic. Biol. Med..

[63] Henriquez-Olguin C., Meneses-Valdes R., Jensen T.E. (2020). Compartmentalized muscle redox signals controlling exercise metabolism – current state, future challenges. Redox Biol.

[64] Yakes F.M., Van Houten B. (1997). Mitochondrial DNA damage is more extensive and persists longer than nuclear DNA damage in human cells following oxidative stress. Proc. Natl. Acad. Sci. U.S.A..

[65] Ballinger S.W., Van Houten B., Conklin C.A., Jin G.F., Godley B.F. (1999). Hydrogen peroxide causes significant mitochondrial DNA damage in human RPE cells. Exp. Eye Res..

[66] Han Y., Chen J.Z. (2013). Oxidative stress induces mitochondrial DNA damage and cytotoxicity through independent mechanisms in human cancer cells. BioMed Res. Int..

[67] Goncalves R.L.S., Quinlan C.L., Perevoshchikova I.V., Hey-Mogensen M., Brand M.D. (2015). Sites of superoxide and hydrogen peroxide production by muscle mitochondria assessed ex vivo under conditions mimicking rest and exercise. J. Biol. Chem..

[68] Ng L.F., Gruber J., Cheah I.K., Goo C.K., Cheong W.F., Shui G., Sit K.P., Wenk M.R., Halliwell B. (2014). The mitochondria-targeted antioxidant MitoQ extends lifespan and improves healthspan of a transgenic Caenorhabditis elegans model of Alzheimer disease. Free Radic. Biol. Med..

[69] Dare A.J., Bolton E.A., Pettigrew G.J., Bradley J.A., Saeb-Parsy K., Murphy M.P. (2015). Protection against renal ischemia-reperfusion injury in vivo by the mitochondria targeted antioxidant MitoQ. Redox Biol.

[70] Hu Q., Ren J., Li G., Wu J., Wu X., Wang G., Gu G., Ren H., Hong Z., Li J. (2018). The mitochondrially targeted antioxidant MitoQ protects the intestinal barrier by ameliorating mitochondrial DNA damage via the Nrf2/ARE signaling pathway. Cell Death Dis..

[71] Brand R.M., Wipf P., Durham A., Epperly M.W., Greenberger J.S., Falo L.D. (2018). Targeting mitochondrial oxidative stress to mitigate UV-induced skin damage. Front. Pharmacol..

[72] Robb E.L., Hall A.R., Prime T.A., Eaton S., Szibor M., Viscomi C., James A.M., Murphy M.P. (2018). Control of mitochondrial superoxide production by reverse electron transport at complex I. J. Biol. Chem..

[73] Muller F.L., Liu Y., Van Remmen H. (2004). Complex III releases superoxide to both sides of the inner mitochondrial membrane. J. Biol. Chem..

[74] Schlame M., Greenberg M.L. (2017). Biosynthesis, remodeling and turnover of mitochondrial cardiolipin. Biochim. Biophys. Acta Mol. Cell Biol. Lipids.

[75] Fernández-Moriano C., González-Burgos E., Gómez-Serranillos M.P. (2017). Lipid peroxidation and mitochondrial dysfunction in alzheimer's and Parkinson's diseases: role of natural products as cytoprotective agents. Neuroprotective Nat. Prod. Clin. Asp. Mode Action..

[76] Wong-Ekkabut J., Xu Z., Triampo W., Tang I.M., Tieleman D.P., Monticelli L. (2007). Effect of lipid peroxidation on the properties of lipid bilayers: a molecular dynamics study. Biophys. J..

[77] Ademowo O.S., Dias H.K.I., Burton D.G.A., Griffiths H.R. (2017). Lipid (per) oxidation in mitochondria: an emerging target in the ageing process?. Biogerontology.

[78] Fogarty M.C., Hughes C.M., Burke G., Brown J.C., Trinick T.R., Duly E., Bailey D.M., Davison G.W. (2011). Exercise-induced lipid peroxidation: implications for deoxyribonucleic acid damage and systemic free radical generation. Environ. Mol. Mutagen..

[79] Bhattacharya A., Muller F.L., Liu Y., Sabia M., Liang H., Song W., Jang Y.C., Ran Q., Van Remmen H. (2009). Denervation induces cytosolic phospholipase a 2-mediated fatty acid hydroperoxide generation by muscle mitochondria. J. Biol. Chem..

[80] Pond S.M., Tozer T.N. (1984). First-pass elimination basic concepts and clinical consequences. Clin. Pharmacokinet..

[81] St-Pierre J., Buckingham J.A., Roebuck S.J., Brand M.D. (2002). Topology of superoxide production from different sites in the mitochondrial electron transport chain. J. Biol. Chem..

[82] Williamson J., Hughes C.M., Davison G.W. (2018). Exogenous plant-based nutraceutical supplementation and peripheral cell mononuclear DNA damage following high intensity exercise. Antioxidants.

[83] Fisher-Wellman K., Bloomer R.J. (2009). Acute exercise and oxidative stress: a 30 year history. Dyn. Med..

[84] de Moraes M.S., Guerreiro G., Sitta A., de Moura Coelho D., Manfredini V., Wajner M., Vargas C.R. (2020). Oxidative damage in mitochondrial fatty acids oxidation disorders patients and the in vitro effect of l-carnitine on DNA damage induced by the accumulated metabolites. Arch. Biochem. Biophys..

[85] Hayashi G., Cortopassi G. (2015). Oxidative stress in inherited mitochondrial diseases. Physiol. Behav..

[86] Liguori I., Russo G., Curcio F., Bulli G., Aran L., Della-Morte D., Gargiulo G., Testa G., Cacciatore F., Bonaduce D., Abete P. (2018). Oxidative stress, aging, and diseases. Clin. Interv. Aging.

[87] Wiegman C.H., Michaeloudes C., Haji G., Narang P., Clarke C.J., Russell K.E., Bao W., Pavlidis S., Barnes P.J., Kanerva J., Bittner A., Rao N., Murphy M.P., Kirkham P.A., Chung K.F., Adcock I.M., Brightling C.E., Davies D.E., Finch D.K., Fisher A.J., Gaw A., Knox A.J., Mayer R.J., Polkey M., Salmon M., Singh D. (2015). Oxidative stress-induced mitochondrial dysfunction drives inflammation and airway smooth muscle remodeling in patients with chronic obstructive pulmonary disease. J. Allergy Clin. Immunol..

[88] Bryant R.J., Ryder J., Martino P., Kim J., Craig B.W. (2003). Effects of vitamin E and C supplementation either alone or in combination on exercise-induced lipid peroxidation in trained cyclists. J. Strength Condit Res..

[89] Bloomer R.J., Goldfarb A.H., McKenzie M.J. (2006). Oxidative stress response to aerobic exercise: comparison of antioxidant supplements. Med. Sci. Sports Exerc..

[90] Tauler P., Aguiló A., Gimeno I., Fuentespina E., Tur J.A., Pons A. (2006). Response of blood cell antioxidant enzyme defences to antioxidant diet supplementation and to intense exercise. Eur. J. Nutr..

[91] Goldfarb A.H., McKenzie M.J., Bloomer R.J. (2007). Gender comparisons of exercise-induced oxidative stress: influence of antioxidant supplementation. Appl. Physiol. Nutr. Metabol..

[92] Sakellariou G.K., Pearson T., Lightfoot A.P., Nye G.A., Wells N., Giakoumaki I.I., Griffiths R.D., McArdle A., Jackson M.J. (2016). Long-Term administration of the mitochondria-Targeted antioxidant mitoquinone mesylate fails to attenuate age-related oxidative damage or rescue the loss of muscle mass and function associated with aging of skeletal muscle. Faseb. J..

[93] Bersuker K., Hendricks J., Li Z., Magtanong L., Ford B., Tang P.H., Roberts M.A., Tong B., Maimone T.J., Zoncu R., Bassik M.C., Nomura D.K., Dixon S.J., Olzmann J.A. (2020). HHS Public Access.

[94] Davison G.W., George L., Jackson S.K., Young I.S., Davies B., Bailey D.M., Peters J.R., Ashton T. (2002). Exercise, free radicals, and lipid peroxidation in type 1 diabetes mellitus. Free Radic. Biol. Med..

[95] Kaur P., Radotra B., Minz R.W., Gill K.D. (2007). Impaired mitochondrial energy metabolism and neuronal apoptotic cell death after chronic dichlorvos (OP) exposure in rat brain. Neurotoxicology.

[96] Wani W.Y., Gudup S., Sunkaria A., Bal A., Singh P.P., Kandimalla R.J.L., Sharma D.R., Gill K.D. (2011). Protective efficacy of mitochondrial targeted antioxidant MitoQ against dichlorvos induced oxidative stress and cell death in rat brain. Neuropharmacology.

[97] Ingram K.H., Hill H., Moellering D.R., Hill B.G., Lara-Castro C., Newcomer B., Brandon L.J., Ingalls C.P., Penumetcha M., Rupp J.C., Garvey W.T. (2012). Skeletal muscle lipid peroxidation and insulin resistance in humans. J. Clin. Endocrinol. Metab..

[99] Shaposhnikov S., Frengen E., Collins A.R. (2009). Increasing the resolution of the comet assay using fluorescent in situ hybridization-a review. Mutagenesis.

